# Targeting CRABP-II overcomes pancreatic cancer drug resistance by reversing lipid raft cholesterol accumulation and AKT survival signaling

**DOI:** 10.1186/s13046-022-02261-0

**Published:** 2022-03-08

**Authors:** Shuiliang Yu, Lei Wang, Danian Che, Mei Zhang, Ming Li, Mikihiko Naito, Wei Xin, Lan Zhou

**Affiliations:** 1 Department of Pathology, School of Medicine, Case Western Reserve University, Cleveland, OH USA; 2 Department of Biomedical Engineering, School of Engineering, Case Western Reserve University, Cleveland, OH USA; 3 Biostatistics and Bioinformatics Core Facility, Case Comprehensive Cancer Center, School of Medicine, Case Western Reserve University, Cleveland, OH USA; 4 Division of Biostatistics of the Department of Preventive Medicine, Keck School of Medicine, University of Southern California, Los Angles, California USA; 5 Division of Molecular Target and Gene Therapy Products, National Institute of Health Sciences, Kanagawa, Japan; 6 Department of Pathology, University Hospitals Case Medical Center, Cleveland, OH USA

**Keywords:** Pancreatic cancer, Drug resistance, Cholesterol, Lipid raft, CRABP-II, SNIPER-11

## Abstract

**Background:**

Resistance to standard therapy is a major reason for the poor prognosis of pancreatic ductal adenocarcinoma (PDAC). Developing novel therapy to overcome PDAC drug-resistance is urgently needed. CRABP-II was highly expressed in all PDAC but not expressed in normal pancreatic tissues and chronic pancreatitis. CRABP-II was shown to promote PDAC migration and metastasis while its potential role in promoting PDAC drug-resistance was not known.

**Methods:**

A paired cohort of human primary and relapsing PDAC tissues was assessed for CRABP-II expression by immunohistochemistry. CRISPR/cas9 gene editing was used to establish CRABP-II knockout cell lines and MTT assays were performed to assess gemcitabine sensitivity in vitro. Cleaved caspase-3/PARP blots and Annexin V staining were conducted to detect cell apoptosis. Gene expression microarray, Q-PCR, western blots, Co-IP and RNA-IP were used to study the molecular function of CRABP-II. Sucrose gradient ultracentrifugation was applied to isolate lipid rafts and LC–MS-MS was used to assess cholesterol content. Both subcutaneous CDX models and orthotopic PDX models were established to examine the efficacy of SNIPER-11 and the synergistic effect between SNIPER-11 and gemcitabine in vivo.

**Results:**

A higher expression of CRABP-II was found in relapsing PDAC tissue and was associated with poor prognosis. Gemcitabine-resistant cell lines exhibited increased level of CRABP-II, while CRABP-II knockout resensitized PDAC cells to gemcitabine. Mechanistically, aberrant expression of CRABP-II increased the stability of SREBP-1c mRNA through cooperation with HuR and upregulated the downstream genes of SREBP-1c to favor cholesterol uptake and accumulation in lipid rafts. Increased lipid raft cholesterol accumulation facilitated ATK survival signaling and PDAC drug resistance. The small compound SNIPER-11 treatment effectively induced CRABP-II protein degradation, induced apoptosis, and suppressed tumor growth. Combination of SNIPER-11 and gemcitabine significantly reduced the lipid raft cholesterol content in CDX/PDX and profoundly inhibited tumor progression.

**Conclusions:**

These findings identified CRABP-II as a novel regulator of cholesterol metabolism and suggested that CRABP-II is a selective target for overcoming PDAC drug resistance.

**Supplementary Information:**

The online version contains supplementary material available at 10.1186/s13046-022-02261-0.

## Background

Pancreatic ductal adenocarcinoma (PDAC) is projected to become the second deadliest cancer in USA and Europe during the next 10 years [1]. In the past couple of decades, the outcome of PDAC has not been improved substantially with the overall 5-year survival rate remains dismal around 8% [2]. The rapid development of resistance to standard therapy such as gemcitabine is thought to be a primary reason for this dismal clinic outcome. Drug resistance of PDAC is mediated by different mechanisms, such as gene mutations involved in intracellular signaling and critical metabolic pathways. Although KRAS drives the majority of PDAC cases (85–90%) and is associated with chemo-resistance [3], it has not been effectively targeted for PDAC patients. Thus understanding molecular and cellular basis of drug resistance and identification of novel molecules and pathways that can serve as targets to overcome PDAC drug resistance is urgently needed.

We previously reported that a small lipid binding protein, cellular retinoic acid binding protein II (CRABP-II), is a novel biomarker of PDAC [4]. CRABP-II is overexpressed in all PDAC tumors but is not detected in normal pancreatic tissues and chronic pancreatitis. By forming a hydrophobic binding cavity, CRABP-II binds to retinoic acid (RA) with high affinity and shuttles RA from cytosol to nucleus, thus facilitating the ligation of RAR and enhancing its transcriptional activity [5–7]. In addition to this conventional role as a carrier for RA, CRABP-II has been shown to interact with HuR, one of the best characterized RNA binding proteins. It was suggested that CRABP-II stabilizes some transcripts by cooperating with HuR [8]. We reported that CRABP-II/HuR complex bound to the 3’UTR of interleukin 8 (IL-8) mRNA and enhanced cancer cell migration and invasion through IL-8/MMP-14/MMP-2 pathway by extending IL-8 mRNA stability [9].

Herein, we report that CRABP-II contributes to PDAC drug resistance through regulating the cholesterol accumulation in lipid rafts. Lipid rafts serve as scaffolds for most molecules of cell signal transduction, thus are capable of regulating multiple pathways which control cell proliferation, survival and migration. We found that CRABP-II directly modulates the expression of the sterol regulatory element-binding protein 1c (SREBP-1c), a central lipid metabolic transcription factor by a post-transcriptional machinery. SREBP-1c in turn regulates an array of cholesterol metabolic genes and controls cellular cholesterol biosynthesis, uptake and efflux. As a result, the accumulation of cholesterol promoted by CRABP-II in lipid rafts increased the AKT survival signaling and reduced gemcitabine mediated PDAC apoptosis. Importantly, we show that a CRABP-II degradation inducing compound SNIPER-11 (specific and nongenetic IAP dependent protein Eraser 11) reduced tumor cell membrane cholesterol content and increased tumor apoptosis. When combined with gemcitabine, SNIPER-11 drastically induced tumor regression in both patient-derived xerografts (PDX) and gemcitabine resistant cell line-derived xerografts (CDX). These findings revealed the potential of CRABP-II as a novel therapeutic target for overcoming PDAC drug-resistance.

## Methods

### Cell lines, antibodies and reagents

Human pancreatic cancer cell lines, BxPC3, Capan-1, Panc-1, Panc10.05 and 293 T cells were obtained from ATCC. CRABP-II knockout (CIIKO) and CRISPR negative control lines were established using Panc-1 as the parental line as described [9]. Gemcitabine resistant lines GR2000 and GR4000 were derived from gemcitabine sensitive line BxPC3 by treating the cells with a stepwise increase of gemcitabine. Panc-1, 293 T, CIIKO and negative control cells were maintained in Dulbecco's modified Eagle's medium (DMEM) supplemented with 4.5 g/L glucose, 4.5 g/L L-glutamine, 10% FBS (Gibco) and 100 IU/mL penicillin/streptomycin. BxPC3, GR2000 and GR4000 cells were maintained in RPMI1640 medium supplemented with 4.5 g/L L-glutamine, 1 mM sodium pyruvate, 10% FBS and 100 IU/mL penicillin/streptomycin. All transfections were performed by using lipofectamine 3000 (Invitrogen).

Antibodies used in this study include: CRABP-II mouse mAbs (Millipore, MAB5488), CRABP-II rabbit polyclonal antibody (Proteintech, 10,225–1-AP), HuR (3A2, Santa Cruz, sc-5261), Flotilin-2 (Santa Cruz, sc-28320), GAPDH (Santa Cruz, sc-365062), and Actin (Santa Cruz, sc-1615), anti-Flag M2 mAb (Sigma, F9291), anti-Flag agarose beads (Clontech, #635,686), Ki67 (SP6, ThermoFisher, RM-9106-S0), ADRP (Novus, NB110-40,877), Caspas3 (Cell Signaling, #9662), PARP (Cell Signaling, #9542), AKT (Cell Signaling, #4691), mTOR (Cell Signaling, #2983), S6 (Cell Signaling, #2217), pAKT (S473, Cell Signaling, #9018), pmTOR (Cell Signaling, #5536), pS6 (Cell Signaling, #4858), and pGSK3β (Cell Signaling, #5558).

SNIPER-11 and MV1 were synthesized by Mekoo Biosciences (Morrisville, NC). Other reagents include: retinoic acid, cholesterol, actinomycin D and gemcitabine hydrochloride (USP standard) (Sigma); insulin (ThermoFisher); protein A/G agarose beads (Millipore); BODIPY dyes (ThermoFisher).

### RNA isolation and real time Q-PCR

Total RNA was isolated using Trizol (Invitrogen). 500 ng of RNA was reverse transcribed to cDNA by using iScript Reserve Transcription Supermix (Bio-Rad). Real time PCR were performed with SYBR green on C1000 CFX96 real time system (Bio-Rad). The primers used are listed in Table S1.

### MTT assay, Annexin V staining and BODIPY staining

MTT assay was performed to assess the cell viability upon gemcitabine treatment. BxPC3 cells and gemcitabine resistant GR2000/GR4000 cells were counted by trypan blue staining and seeded into 96-well plates at 5000 cell/well in triplicates. 6 h after seeding, the adhered cells were treated with gemcitabine for 72 h in a series of increasing concentrations from 0 µM to 100 µM. Cell viability was assessed by using Celltiter96 AQueous One Solution Reagent (Promega). For gemcitabine sensitivity comparison between Panc-1 cells and CIIKO cells, all parental Panc-1, CIIKO and CRABP-II re-expressing (CIIOE) cells were counted and seeded into 96-well plates at 3000 cell/well in triplicates. After adhesion, cells were treated with gemcitabine in hormone-depleted medium (Gibco) for 96 h and cell viability was assessed.

To evaluate the early stage cell apoptosis induced by gemcitabine, Annexin V staining was performed following the manufactory description (BD Pharmingen). Briefly, cells were treated with 50 µM of gemcitabine for 24 h. The suspended cells were collected while the adherent cells were shortly trypsinized. Both parts of cells were combined and washed with cold PBS and resuspended in binding buffer at about 1 × 10^6^ cells/mL. 100 µL of cell suspension was stained with 5 µL FITC-Annexin solution and 2 µL Propidium Iodide (PI) for 15 min at 25ºC before FACS analysis on FACSAria™ II flow cytometer (BD).

For neutral lipid content detection, CIIKO and wild type control cells were rinsed with PBS then incubated with 2 µM BODIPY staining solution in PBS at 37ºC for 15 min. After a quick PBS wash, cells were trypsinized to single cell suspension and submitted to FACS analysis.

### Immunoprecipitation (IP) and RNA-immunoprecipitation (RIP)

GR4000 cells were cultured in hormone-depleted media for 48 h and grew to 70–80% confluence. After cold PBS washes, cells were lysed in EBC buffer (50 mM Tris, pH8.0, 120 mM NaCl and 0.05% IGEPAL) supplemented with cocktail proteinase inhibitor (Roche). 1–2 mg of total proteins in cell lysate were mixed with 10 μg of rabbit IgG isotype control or anti-CRABP-II polyclonal antibody (Proteintech) and incubated at 4 °C overnight. Protein A agarose beads were added and incubated for 1 h. Washed beads were boiled in 30 μL of 2 × SDS loading buffer and protein samples were loaded on SDS-PAGE. Separated proteins on PVDF membrane were blotted with anti-HuR antibody.

For RNA immunoprecipitation (RIP), flag-CRABP-II expressing CIIKO cells and empty vector transfected cells were grown in hormone-depleted media for 48 h and followed by ice-cold PBS wash for twice. Cells were collected and lysed in polysome lysis buffer (10 mM HEPES, pH7.0, 100 mM KCl, 5 mM MgCl_2_, 25 mM EDTA, 0.5% IGEPAL) containing 2 mM DTT, RNase OUT (Invitrogen), proteinase inhibitors and 0.2 mg/mL Heparin. After centrifuge, 20 mg cell extract was removed to new 15 ml tubes and diluted in 10 × volumes of NT2 buffer (50 mM Tris, pH7.5, 150 mM NaCl, 1 mM MgCl_2_ and 0.05% IGEPAL). Anti-flag beads were washed, suspended in NT2 buffer with 5% BSA, 0.02 mg/ml heparin, and rotated with diluted cell extract at 4 °C for 4 h. After washes with ice-cold NT2 buffer, the beads and 100 µL of original cell extract (used as input) were mixed with Trizol reagents and centrifuged. RNA in aqueous phase was precipitated by adding isopropanol and RNAase free glycogen (Invitrogen). SREBP-1c and actin messages were assessed by Q-PCR.

### Lipid raft isolation and cholesterol quantification

Sucrose gradient ultracentrifugation was used for membrane fractionation as described before [10]. To quantify the total cholesterol in lipid rafts, the raft enriched fractions were mixed with equal volume of methanol: 12 N HCl (10:1) and 2 volumes of chloroform, centrifuged at 13,000 xg for 10 min at 4ºC. The aqueous phases were removed and the chloroform phases were re-extracted with 1 volume of methanol: 1 N HCl (1:1). After removing aqueous phases, the chloroform was evaporated at 50ºC for 3 h and the dried lipids were dissolved in cholesterol assay buffer by sonication. The total cholesterol was quantified using the Cholesterol Quantitation Kit (Sigma) according to the manufacturer’s instruction.

### Human PDAC collection and immunohistochemistry

Paraffin-embedded blocks of 12 surgically resected, primary PDACs and their relapsing biopsies were collected from the Pathology Archives. Tissue slides were processed using a BenchMark Ultra automated immunostainer (Ventana Medical Systems, Tucson AZ). Slides were deparaffinized, antigen retrieved, and incubated at 37ºC with the primary anti-CRABP-II, or anti-ADRP. Histologic images were obtained with the ScanScope XT digital scanning system (Aperio Technologies, Vista, CA). Cytoplasmic and nuclear immunoreactivity was considered as a positive expression. Immunoreactivity was scored by 2 investigators independently based on the percentage and intensity of positive epithelium cells (intensity: 0, undetectable; 1, weak; 2, moderate; and 3, strong). Score 0 was considered as negative; score 1 or above, as positive.

### PDAC PDX mouse models and SNIPER-11 treatment

Xenografts were established in 6–8 weeks NOD-SCID-gamma (NSG) mice. Briefly, the limited passaged (2–3 passages) PDX tumors were resected from mice and rinsed with cold PBS. Tumors were cut into 1–2 mm^3^ cubes and transplanted to the back of NSG mice subcutaneously. The tumor growth was monitored by measuring the diameters with calipers and the tumor volume was calculated as the flowing formula: volume (mm^3^) = ½ (width * width * length). Dissected tumors will be frozen at -80ºC or fixed in 10% formalin for biochemical and pathology evaluation. For SNIPER-11 treatment, the tumor bearing mice were intraperitoneally (IP) administrated with SNIPER-11 (5 µM or 5.31 mg/kg, every two days, for 3 weeks) or DMSO when the tumors grew to about 100 mm^3^. Mice treated with 5 µM of MV1 or RA were included as controls in some of the experiments.

Orthotopic PDX models were established as reported [11]. In brief, limited passaged PDX tumors were resected from mice and rinsed in serum free RPMI 1640. Tumors were minced into the smallest possible fragments and digested into single-cell suspension with collagenase IV solution (200 U/mL, fresh prepared in RPMI 1640). After digestion, tumor cells were washed with RPMI 1640 and stained through a 70 µm strainer. Red blood cells (RBC) were removed using RBC lysis buffer and the resulting cell solution was washed and stained through a 40 µm strainer. Single cells were collected by centrifuge, counted and resuspended in serum free RPMI 1640 at 2 × 10^6^ cell/100 µL. 50 µL of cell suspension was mixed with Matrigel at 1:1 ratio and injected to pancreas in NSG mice. Tumor growth was assessed by MRI imaging at the Case Center for Imaging Research (CCIR). Starting from 4–5 weeks after implantation, tumor bearing mice were IP treated with SNIPER-11 (5 µM, every two days), gemcitabine (20 mg/kg weekly) or a combination of SNIPER-11 and gemcitabine for 3 weeks, DMSO was used as negative control.

### Gemcitabine resistant CDX models and treatment

Xenografts were established in 6–8 weeks nude mice. Briefly, GR4000 gemcitabine resistant cells were mixed with Matrigel at 1:1 ratio, and subcutaneously injected (1 × 10^6^ cells/injection) into the lower flank of the mice at both sides. Three weeks after implantation, tumor bearing mice were IP treated with SNIPER-11 (5 µM, every two days), gemcitabine (20 mg/kg weekly) or a combination of SNIPER-11 and gemcitabine for 3 weeks, DMSO was used as negative control. Tumors in each treatment group were resected and weighed. Tumors in each treatment group were combined and digested to single cells. Tumor cells were enriched by depleting infiltrating immune cells using anti-CD45 microbeads (Miltenyi Biotec, 130–052-301). Enriched tumor cells were submitted to lipid raft fraction and cholesterol assays.

### Statistics

The Kaplan–Meier method was used to determine overall survival with respect to CRABP-II expression based on the data from Kaplan–Meier Plotter database. The CRABP-II or ADRP staining comparison between primary tumors and relapsing tumors were conducted with a paired sample t-test. Two-way ANOVA was used for tumor growth comparison with respect to different treatments. Student’s t-test was used for other two means comparison. All the statistics were performed using Graphpad Prism software or Excel and *p* value < 0.05 was regarded as significant difference.

## Results

### High expression of CRABP-II is associated with poor survival outcome in PDAC

**Fig. 1:**
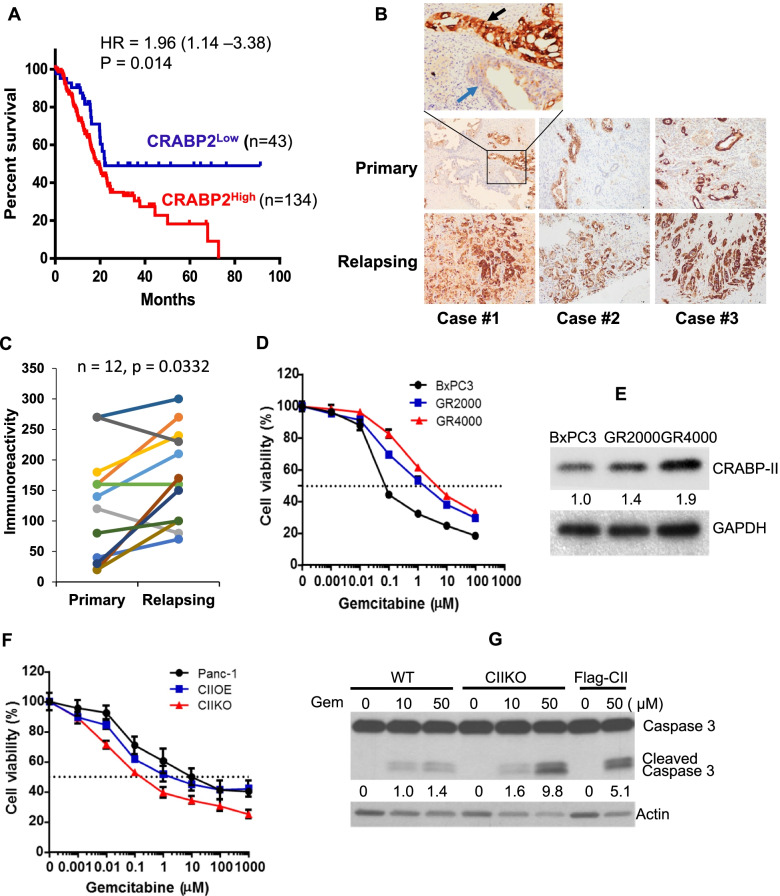
High expression of CRABP-II is associated with poor prognostics and drug resistance in human PDAC. **A** Kaplan–Meier overall survival curves for PDAC patients based on CRABP-II levels. Data shown here are from Kaplan–Meier Plotter database. **B, C** CRABP-II expression in paired primary and relapsing PDAC (*n* = 12) assessed by immunochemistry. All patients received gemcitabine-based chemotherapy after surgery. Immunoreactivity in (**C**) was calculated by multiplying the percentage of positive epithelium cells and the score of staining intensity (intensity: 0, undetectable; 1, weak; 2, moderate; and 3, strong). Paired sample t-test was used to determine the difference between primary group and relapsing group. Up panel in (**B**): black arrow denotes the poorly differentiated tumor; blue arrow denotes the well differentiated tumor. **D, E** Establishment of gemcitabine resistant cell lines using BxPC3 as parental line. Gemcitabine sensitivities of GR2000, GR4000 and BxPC3 cells were assessed by MTT assay (**D**). CRABP-II levels in these lines were detected by western blots (**E**). **F** Gemcitabine sensitivity comparison of Panc-1, CRABP-II knockout (CIIKO) and CRABP-II re-expressing (CIIOE) cells by MTT assays. **G** Gemcitabine induced cell apoptosis assessed by cleaved caspase-3 blots

In our previous study, we found that all PDAC tumors display overexpression of CRABP-II, while neither normal pancreatic parenchyma or ductal epithelium, nor chronic pancreatitis express CRABP-II [4]. Notably higher level of CRABP-II in metastatic lymph nodes was found when compared to primary tumors, suggesting that CRABP-II may mediate intrinsic chemo-resistance in PDAC tumors. To investigate the role of CRABP-II in PDAC progression and drug-resistance, we examined the patient survival dataset in Kaplan–Meier plotter database. Patients with higher CRABP-II expression levels have significantly reduced overall survival and have shortened median survival from 22.03 months (low expression) to 18.93 months (high expression) (Fig. 1a). We then compared CRABP-II levels in recurrent tumor sections from 12 PDAC patients and their respective primary tumor sections. All these patients received gemcitabine-based chemotherapy after surgery. Overall, relapsing tumors show poor differentiation and higher CRABP-II expression by immunohistochemistry (Fig. 1b&1c). Further, in primary tumors, we found that CRABP-II expression was higher in poorly differentiated tumor cells than in well differentiated cells (Fig. 1b, black and blue arrows in case #1, top panel). Because poor differentiation is associated with poor outcome and increased chemo-resistance [12], these observations indicate that elevated CRABP-II expression may contribute to PDAC drug-resistance and increased risk of recurrence.

### Deletion of CRABP-II sensitized PDAC cells to gemcitabine induced apoptosis

To study the role of CRABP-II in the regulation of PDAC drug-resistance, we established two gemcitabine resistant cell lines, GR2000 and GR4000, through multiple rounds of gemcitabine selection from the gemcitabine sensitive cell line BxPC3. As a result, the gemcitabine IC50 of BxPC3 cells was increased from 75 nM to 1.8 µM in GR2000 and 4.7 µM in GR4000, respectively (Fig. 1d). Consistently, the CRABP-II level was also elevated by 1.4-fold and 1.9-fold in GR2000 and GR4000, respectively, when compared to the parental cells (Fig. 1e). In comparison to BXPC3, Panc-1 cells have much higher CRABP-II expression and are more gemcitabine resistant than BxPC3 cells (Fig. S1). Deletion of CRABP-II in Panc-1 cells by CRSPER/Cas9 (Fig. S2) remarkably decreased gemcitabine IC50 from ~ 10 µM to ~ 0.2 µM **(**Fig. 1f). Accordingly, CRABP-II knockout (CIIKO) cells exhibited enhanced cell apoptosis after gemcitabine treatment (Fig. 1g; Fig. S3). To test the specific effect of CRABP-II on apoptosis induction, we re-expressed a flagged-CRABP-II in CIIKO cells (Fig. S2). Despite that the restored expression of flagged-CRABP-II was at about 20% of the endogenous CRABP-II in parental cells, it was capable of partially reversing the gemcitabine induced apoptosis (Fig. 1g; Fig. S3) and IC50 decrease (Fig. 1f). These observations demonstrated that CRABP-II is involved in the regulation of PDAC chemo-resistance.

### CRABP-II altered lipid metabolism and enhanced cholesterol metabolic gene expression

**Fig. 2:**
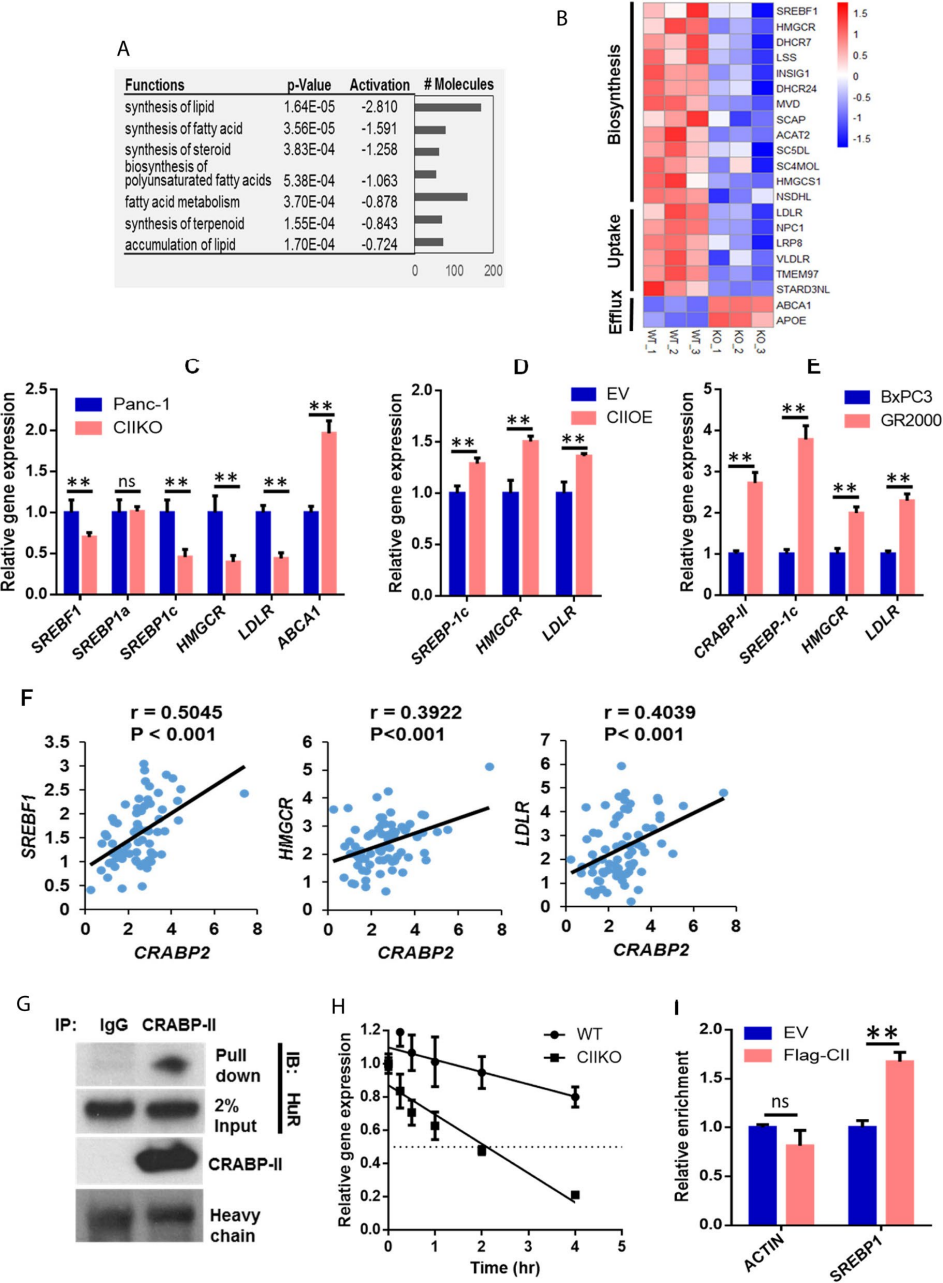
CRABP-II regulates cholesterol metabolic genes expression through cooperation with HuR. (**A**) Molecular and cellular function analysis by IPA software (Qiagen) based on gene expression microarray profiling. The altered lipid synthesis and accumulation functions upon CRABP-II knockout were listed. (**B**) Heat map of altered cholesterol metabolic genes. (**C, D, E**) Cholesterol metabolic genes expression assessed by Q-PCR. (**F**) Correlation between cholesterol metabolic genes and CRABP-II expression in human pancreatic cancer specimens by Pearson’s product-moment correlation coefficient analysis (PPMCC). Data shown here are combination of Pei Pancreas and Badea Pancrease datasets (*n* = 75) from Oncomine. (**G**) Interaction between CRABP-II and HuR identified by co-immuprecipitation (co-IP). GR4000 cell lysis was incubated with anti-CRABP-II rabbit polyclonal antibody and the pull down proteins were separated and blotted with anti-HuR mouse monoclonal antibody. (**H**) Half-life of SREBP-1c mRNA assessed by actinomycin D treatment following with Q-PCR. (**I**) RNA-immunoprecipitation (RIP). The down pulled SREBP-1c mRNA from flagged-CRABP-II transfected CIIKO cells and empty vector transfected cells were assessed by Q-PCR. The actin mRNA was used as control. The experiment was repeated three times and the error bars present standard deviation (SD). **, p < 0.01

To understand the molecular mechanism by which CRABP-II regulates PDAC survival and chemo-resistance, we performed gene expression array analysis. Total RNA was isolated from CIIKO cells and control Panc-1 cells that had been cultured in hormone-depleted media for 3 days to eliminate effect of retinoic acid. We found that 1895 genes were down-regulated while 1478 genes were up-regulated upon CRABP-II deletion. Ingenuity Pathway Analysis (IPA) revealed that, consistent with cell culture studies, both apoptosis and cell death were increased in CIIKO cells [9]. Notably, functions of lipid metabolism including both lipid synthesis and accumulation are profoundly down-regulated by CRABP-II knockout (Fig. 2a). A total of 169 lipogenic genes and 73 lipid accumulation-related genes changed their expression in CIIKO cells. A further examination into the gene list revealed that cholesterol metabolic genes involved in the intracellular cholesterol biosynthesis, uptake and efflux are a main cluster of genes whose expression were altered (Fig. 2b). Among these, the sterol regulatory element-binding protein 1 (SREBP-1) gene, *SREBF1*, was significantly decreased in CIIKO cells. SREBP-1 is a central player in lipid metabolic modulation. *SREBF1* gene transcribes two isoforms, SREBP-1a and SREBP-1c [13]. Quantitative PCR (Q-PCR) further revealed that *SREBP-1c*, but not *SREBP-1a*, is downregulated by 2 folds in CIIKO cells (Fig. 2c). SREBP-1c is the predominant isoform expressed in most tissues whereas SREBP-1a is highly expressed in intestinal epithelial, heart, macrophage and bone marrow dendritic cells [14]. When expressed at a normal level, SREBP-1c mainly regulates gene expression required for fatty acid synthesis. However, it activates genes for both cholesterol synthesis and intake when expressed at higher level in tumors [13]. The downstream targets of SREBP-1c include cholesterol biosynthetic genes including *HMGCR*, *HMGCS1*, *MVD*, *INSIG*, and cholesterol import genes such as *LDLR*, *TMEM97* [13, 15]. SREBP-1c also negatively regulates the expression of cholesterol efflux regulatory protein ABCA1 through activating *miR-33* expression to enhance cholesterol intracellular accumulation [16]. In line with this, both our microarray and Q-PCR results showed drastically decreased expression of *HMGCR* and *LDLR*, while *ABCA1* expression was increased 2 folds in CIIKO cells (Fig. 2c). Corroborating these findings with CRABP-II deletion, restoring CRABP-II expression in CIIKO cells rescued the loss of *SREBP-1c*, *HMGCR* and *LDLR* (Fig. 2d). Moreover, as the expression of CRABP-II goes up in gemcitabine resistant lines, the expression levels of *SREBP-1c*, *HMGCR* and *LDLR* were all up-regulated (Fig. 2e). Aside from the in vitro discoveries, a significant positive correlation between *CRABP2* and *SREBF1* was found in the Oncomine human PDAC RNA array database, and similar correlations were identified between *CRABP2* and *LDLR* or *HMGCR* in human PDACs (Fig. 2f). Collectively, these findings suggest that CRABP-II is an upstream regulator of PDAC cholesterol metabolism through upregulating SREBP-1c and its downstream genes.

CRABP-II can directly regulate gene expression through an RA-dependent mechanism, or alternatively, contribute to the post-transcriptional regulation of gene expression by cooperating with HuR in an RA independent manner [8]. When bound to the AU-rich element (ARE) in the 3’UTRs of mRNAs, HuR protects the transcripts from degradation [17]. The association with CRABP-II (Fig. 2g) increases the affinity of HuR towards its targeting mRNAs, resulting in the up-regulation of mRNA stability and translational efficiency [8, 9]. Since RA treatment is not able to activate SREBP-1c expression or HMGCR and LDLR expression in Panc-1 cells (data not shown), we examined if the CRABP-II/HuR complex is responsible for the elevated expression of SREBP-1c in PDAC cells. Sequence analysis revealed a putative ARE at 4241–4245 nt in the 3’UTR region of SREBP-1c mRNA (Fig. S4). By assessing the half-life of SREBP-1c mRNA using the transcriptional inhibitor actinomycin D, we found that CRABP-II knockout shortened the half-life of SREBP-1c mRNA from 10.3 h to 2.2 h (Fig. 2h). Furthermore, RNA immunoprecipitation (RIP) analysis revealed that CRABP-II was enriched at the ARE site of SREBP-1c 3’UTR, but not at the ARE site of β-Actin mRNA which is also a target of HuR [18] (Fig. 2i). In comparison, the half-life of LDLR messenger was not affected by CRABP-II knockout (data not shown), indicating that the reduction of LDLR in CIIKO cells is secondary to the downregulation of SREBP-1c.

### Lack of CRABP-II decreased lipid raft cholesterol content and attenuated AKT survival signaling

**Fig. 3:**
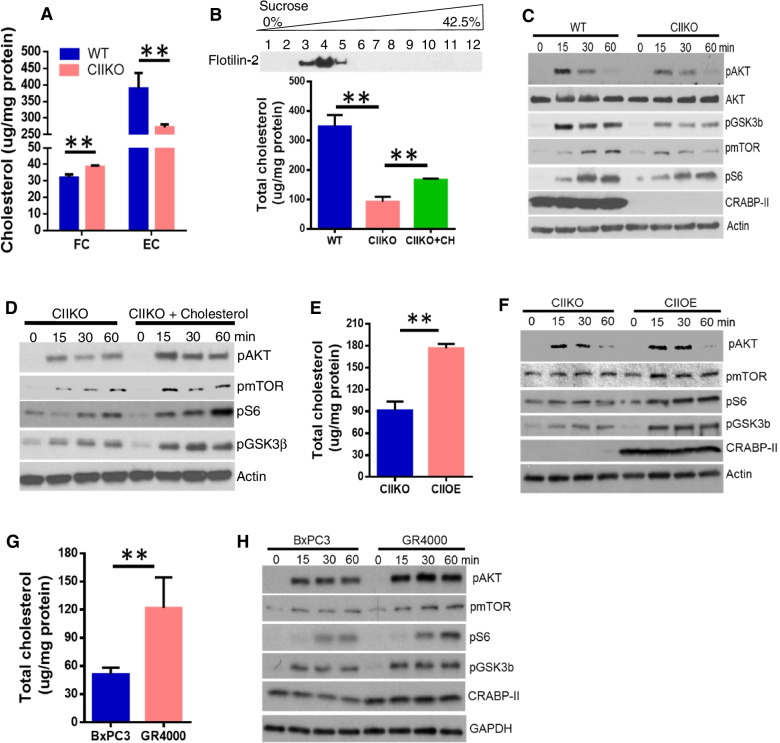
CRABP-II knockout decreased lipid raft cholesterol accumulation and AKT survival signaling. (**A**) Comparison of cholesterol content in CRABP-II knockout cells and in wild type control Panc-1 cells. The free cholesterol (FC) and esterified cholesterol (EC) were assessed by liquid chromatography with tandem mass spectrometry (LC–MS-MS) and normalized to total protein. (**B**) Comparison of lipid raft cholesterol storage in wild type cells and CRABP-II knockout cells. The membrane lipid raft fractions were isolated by sucrose gradient ultracentrifuge (up panel) and the total cholesterol was assessed using cholesterol assay kit and normalized to total protein. The green bar presents the CIIKO cells pretreated with 1 mM cholesterol in media. (**C, D**) AKT activation assessed by western blots. Panc-1, CIIKO and cholesterol pretreated CIIKO cells were incubated with 100 nM insulin for 0, 15, 30 and 60 min, cells were immediately lysed. (**E, F**) Rescuing the loss of raft-cholesterol and AKT activation in CIIKO cells by re-expression of CRABP-II. (**G, H**) Increased raft-cholesterol content and AKT activation in gemcitabine resistant cell lines. All these experiments were repeated at least three times and the error bars present SD. **, *p* < 0.01

Because our gene expression array showed that CRABP-II in PDAC cells activated many cholesterol metabolic genes (Fig. 2b), we sought to assess the cholesterol content in the parental cancer cells and the CIIKO cells by BODIPY staining and flow cytometry analysis (Fig. S5). BODIPY is a fluorescent dye which binds to cholesterol ester and triacylglycerol, the major compositions of neutral lipids, in cell membranes and lipid droplets. We found about 40% less storage of neutral lipids in CIIKO cells compared to Panc-1 parental cells. We then evaluated the alternation of cholesterol fractions in Panc-1 cells following CRABP-II deletion by liquid chromatography with tandem mass spectrometry (LC–MS-MS). We found that CRABP-II knockout reduced the esterified cholesterol fraction by 30–40% but slightly increased the free cholesterol content (Fig. 3a). Consistently, staining of a lipid droplet marker, adipose differentiation-related protein (ADRP), in paired tumors revealed a significant increase in the relapsing tumors than in the corresponding primary tumors (Fig. S6).

Cholesterol comprises a major part of mammalian cell membranes, representing about 30% of the lipid bilayer on average. Cholesterol preferentially distributes to lipid rafts, a specialized mircodomain of plasma membranes which enriches 3 to fivefold the amount of cholesterol found in the surrounding bilayer [19]. Acting as the dynamic assemblies of proteins and lipids, lipid rafts harbor various receptors and regulatory molecules. Among these, the serine/threonine kinase AKT functions as a central node of signaling cascades promoting cell survival in cancer cells. When activated by EGF or insulin, AKT can be phosphorylated by PI3K/PDK1 or mTORC2, and in turn regulates a wide range of downstream targets including mTORC1/S6, GSK3, IKK and Bad which control cell proliferation, survival, motility and metabolism. Most of the survival signaling molecules including PI3K, PDK1, AKT and mTOR have been associated with lipid rafts in cancer cells [20]. Herein, we examined the lipid rafts in PDAC cells and the associated key signaling pathways in CIIKO cells. We isolated lipid rafts using sucrose gradient ultracentrifugation and determined the total raft-cholesterol content (Fig. 3b). When compared to the parental cells, the amount of raft-cholesterol was reduced by 75% in CIIKO cells. Not surprisingly, the activation of AKT and its downstream signaling including the phosphorylation of mTOR, S6 and GSK3β were decreased upon CRABP-II deletion (Fig. 3c). The reductions of raft-cholesterol and the suppression of AKT signaling in CIIKO cells were partially reversed by the supplement of extra cholesterol in culture media (Fig. 3b&3d) or the re-expression of CRABP-II (Fig. 3e&3f). Consistently, we found a twofold increase of total raft-cholesterol and an augmentation of AKT activation accompanying increased CRABP-II expression in gemcitabine resistant lines when compared to the parental BxPC3 cells (Fig. 3g&3 h).

Taken together, these observations demonstrated that the highly expressed CRABP-II promotes PDAC drug-resistance through stabilizing SREBP-1c mRNA and the up-regulation of cholesterol metabolic genes, which leads to the elevation of the lipid raft cholesterol accumulation and the AKT survival signaling.

### SNIPER-11 induced CRABP-II degradation and suppressed PDX tumor growth

**Fig. 4:**
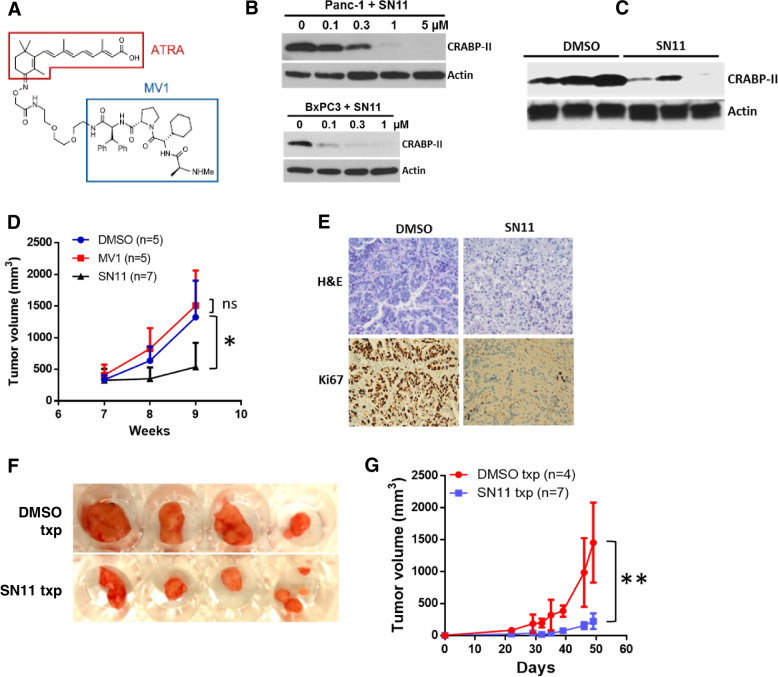
SINPER-11 treatment induced CRABP-II degradation and suppressed PDX tumor growth. (**A**) Molecular formula of SNIPER-11. (**B**) Degradation of CRABP-II induced by SNIPER-11 in Panc-1 and BxPC3 cell culture. (**C**) Degradation of CRABP-II by SNIPER-11 in PDX tumors. (**D**) SNIPER-11 inhibited PDX tumor growth. Limited passaged PDX tumors were digested to single cell suspension and orthotopically injected into pancreas of NSG mice (1 × 10^6^ cell / injection). Tumor bearing mice were treated with 5 µM SNIPER-11 or MV1 every two days for two weeks at 7 weeks after implantation and DMSO as control. Tumor size was assessed by MRI imaging (Fig. s7). Two-way ANOVA was used to compare tumor growth between different treatments and the error bars represent SD. *, *p* < 0.05. (**E**) H&E and Ki67 staining of SNIPER-11 or DMSO treated tumor specimens. (**F, G**) The renewal of SNIPER-11/DMSO treated tumors. SNIPER-11 or DMSO treated tumors were secondarily transplanted into subcutaneous cavity of NSG mice. Tumor size was monitored and the tumor volume = ½ (width x width x length). The tumor growth was compared using two-way ANOVA (**, *p* < 0.01)

The aberrant CRABP-II expression in PDAC indicates that CRABP-II may serve as a specific target for PDAC therapy. A retinoic acid derived compound, SNIPER-11 (specific and nongenetic IAP dependent protein Eraser 11) is a potential CRABP-II-targeted drug for PDAC chemotherapy. SNIPER-11 consists of two arms, all-trans RA and MV1 (Fig. 4a), that serves as the high-affinity ligands for CRABP-II and the cellular inhibitor of apoptosis protein 1 (cIAP1), respectively. SNIPER-11 has been shown to induce cIAP1-mediated ubiquitination and proteasome degradation of CRABP-II in neuroblastoma and breast cancer cells [21]. We first tested SNIPER-11 in PDAC cells in vitro and found it effectively induced CRABP-II degradation in both Panc-1 and BxPC3 cells (Fig. 4b). We then examined the in vivo delivery and the anti-tumor effect of SNIPER-11 in PDX mouse models. The tumor bearing mice received either SNIPER-11 or DMSO, RA, MV1 as described in Methods. We found that SNIPER-11 effectively induced the CRABP-II degradation in PDX tumors (Fig. 4c) and significantly inhibited tumor growth (Fig. 4d; Fig. S7). In contrast, neither MV1 nor RA altered tumor growth significantly (Fig. S8). In addition, tumors from animals receiving SNIPER-11 had much lower Ki67 activity but more necrosis compared to the tumors from animal treated with DMSO (Fig. 4e), suggesting that suppressing CRABP-II indeed impaired PDX cell proliferation and promoted tumor apoptosis in vivo. To study if SNIPER-11 has sustained anti-tumor effect, tumors from animals receiving SNIPER-11 or DMSO (controls) were subcutaneously re-planted into another group of NSG mice. While control tumors showed robust growth upon re-planting, SNIPER-11 treated tumors were much smaller upon re-planting (Fig. 4f&4 g), indicating that targeting CRABP-II in PDAC also suppressed cancer cell stemness. To examine the drug toxicity of SNIPER-11, liver and the CRABP-II expressing tissues including skin and esophagus were resected from SNIPER-11 treated mice. No obvious drug toxicity was found associated with the dose used (Fig. S9).

### SNIPER-11 synergized with gemcitabine to suppress PDAC progression

**Fig. 5:**
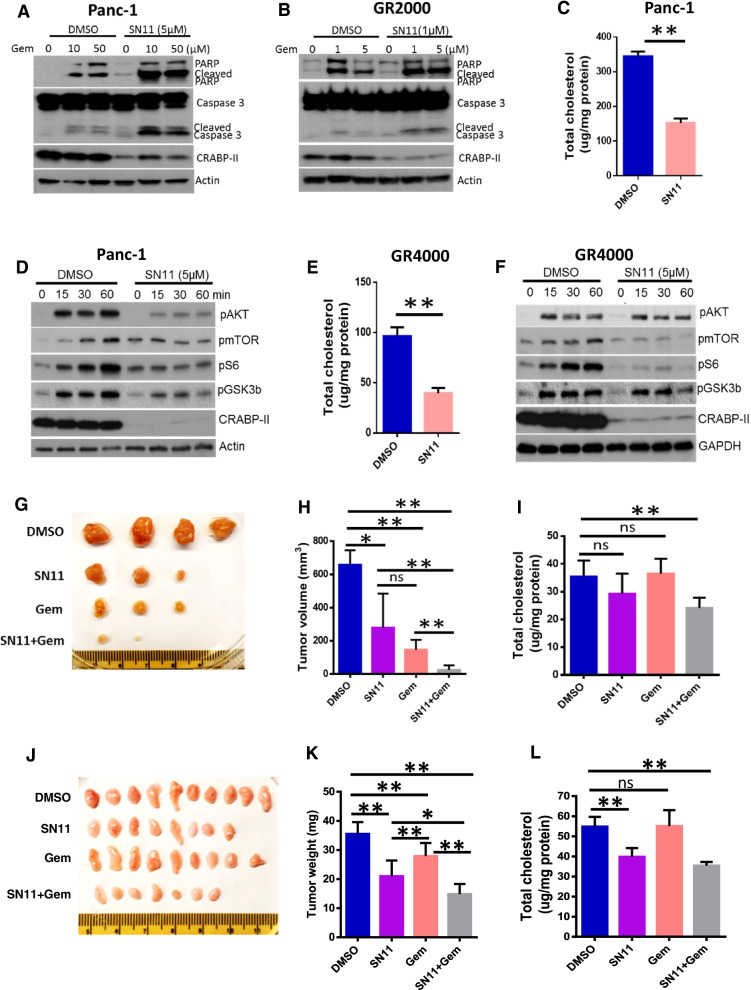
SNIPER-11 enhanced the efficacy of gemcitabine in PDAC. (**A, B**) SNIPER-11 increased gemcitabine induced Panc-1 or GR2000 cell apoptosis. Cells were treated with the combination of SNIPER-11 and gemcitabine for 72 h and cleaved caspase-3/cleaved PARP were detected by western blots. (**C**, **D, E, F**) SNIPER-11 treatment reduced raft-cholesterol accumulation and AKT activation. Panc-1 cells (**C, D**) and GR4000 cells (**E, F**) were treated with 5 µM SNIPER-11 for 48 h, then the lipid raft cholesterol was assessed using cholesterol assay kit. As to AKT signaling, after 48 h treatment with 5 µM SNIPER-11, the starved cells were stimulated with 100 nM insulin for different time as denotes. (**G, H, I**) The synergistic effect between SNIPER-11 and gemcitabine in PDX. 1 × 10^6^ PDX single cells were orthotopically injected to pancreas of NSG mice. Mice were grouped and received DMSO, SNIPER-11, gemcitabine or the combination of SNIPER-11 and gemcitabine at 4 weeks after transplantation as described in Materials and Methods. After 3 weeks treatment, tumors were resected and analyzed. Tumor volume = ½ (width x width x length) (**H**). Tumors were further digested to single cells and lipid raft fraction of these tumor cells were isolated and submitted to cholesterol assays (**I**). (**J, K, L**) The synergistic effect between SNIPER-11 and gemcitabine in gemcitabine-resistant CDX. 1 × 10^6^ GR4000 cells were subcutaneously injected to the lower flank of nude mice. Mice were grouped and received DMSO, SNIPER-11, gemcitabine or the combination of SNIPER-11 and gemcitabine at 3 weeks after implantation. After 3 weeks of treatment, tumors were resected (**J**) and weighed (**K**). Tumors were combined and digested to single cells. After depletion of tumor-infiltrating immune cells, the lipid raft fraction of enriched tumor cells was isolated and submitted to cholesterol assays (**L**). The mean comparison between different groups by student t-test. *, *p* < 0.05; **, *p* < 0.01

Our in vitro data has shown that deletion of CRABP-II re-sensitized Panc-1 cells to gemcitabine induced apoptosis (Fig. 1f&1 g; Fig. S3). We further asked if SNIPER-11 is able to enhance the sensitivity of PDAC tumors to gemcitabine. We first examined the synergistic effect between SNIPER-11 and gemcitabine in PDAC cells. Panc-1 and the gemcitabine-resistant GR2000 cells were treated with gemcitabine alone or gemcitabine combined with SNIPER-11 for 3 days, and cell apoptosis was assessed by blotting with cleaved caspase 3/PARP (Fig. 5a&5b). We found that the addition of SNIPER-11 not only decreased the level of CRABP-II, but also enhanced the cleavage of both caspase 3 and PARP by gemcitabine. We further scaled the lipid raft-cholesterol content in SNIPER-11 treated cells and found that, in agreement with our observations in CIIKO cells, SNIPER-11 treatment decreased raft-cholesterol accumulation by more than 50% in both Panc-1 and GR4000 cells (Fig. 5c&5e). Consistently, SNIPER-11 suppressed AKT survival signaling in both Panc-1 (Fig. 5d) and gemcitabine-resistant BxPC3 (Fig. 5f).

To test the synergistic effect of SNIPER-11 with gemcitabine in vivo, orthotopic PDX bearing mice were treated with SNIPER-11, gemcitabine or with both gemcitabine and SNIPER-11. Compared to control DMSO treatment, SNIPER-11 alone, gemcitabine alone or compound treatment all reduced tumor growth. However, compound treatment showed profound suppression of tumor growth and even complete tumor elimination in some cases (Fig. 5g&5 h). Further, while mice receiving DMSO or gemcitabine presented multiple liver metastasis, mice treated with SNIPER-11 alone or with compound treatment had fewer or no metastasis (Fig. S10). This is in line with our previous finding that CRABP-II regulates PDAC cell migration and invasion [9]. Finally, the raft-cholesterol content in PDX cells was decreased about 30% by treatment with SNIPER-11 and gemcitabine, while the raft-cholesterol content in gemcitabine-treated tumors was not much different from control tumors (Fig. 5i). In comparison, SNIPER-11 alone did not significantly reduce the raft-cholesterol in PDX cells. This is likely caused by the presence of stromal cells and tumor-infiltrating immune cells in PDX tissue that blunt the cholesterol reduction in tumor cells.

We noticed that in the PDX model we established, treatment with gemcitabine alone significantly inhibited tumor growth, suggesting that this PDX is rather sensitive to gemcitabine. To better assess the synergistic effect of SNIPER-11 with gemcitabine on drug-resistant PDAC, we established the gemcitabine resistant xenografts with GR4000 resistant cell line. Compared to control treatment, gemcitabine alone slightly reduced tumor volume. The compound treatment, however, markedly inhibited tumor growth or eliminated tumor in the resistant model (Fig. 5j&5 k). To assess if SNIPER11 treatment indeed reduces raft-cholesterol in PDAC, we enriched tumor cells by depleting tumor-infiltrating immune cells. As expected, both SNIPER-11 and compound treatment reduced the raft-cholesterol content in CDX cells by 30–40% (Fig. 5L**)**. Collectively, these findings support the notion that SNIPER-11 enhances PDAC sensitivity to gemcitabine by inducing CRABP-II-mediated lipid-raft cholesterol accumulation and suppressing CRABP-II mediated survival signaling.

## Discussion

Accelerated influx and endogenous biosynthesis of cholesterol has been regarded as a hallmark of cancer development and are involved in chemotherapy resistance of various malignancies including PDAC [22]. As a major component of cell membranes, cholesterol enriches in the special microdomain of plasma membranes known as lipid rafts. Lipid rafts serve as scaffolds for most molecules of cell signal transduction, and therefore regulate multiple pathways which control cell proliferation, survival and migration. Lowering cholesterol content in lipid rafts of cancer cells with cholesterol synthesis inhibitor statins has been shown to inhibit cell survival mediated by the AKT serine/threonine kinase and induce prostate cancer cell apoptosis [23]. Direct cholesterol membrane depletion using methyl-β-cyclodextrin (MβCD) induced cancer cell apoptosis and sensitized melanoma to tamoxifen [24]. In PDAC, statin treatment potentiated the effect of chemotherapy and improved both median survival and overall survival in PDAC patients [25–28]. In addition, disruption of cholesterol uptake by silencing cholesterol transporter LDLR sensitized PDAC cells to gemcitabine [29]. Therefore, targeting lipid raft cholesterol attracts increasing attention as a cancer therapeutic strategy. However, despite that both statins and MβCD are currently being tested in ongoing clinical trials for cancer treatment, long-term cholesterol lowering by either statins or MβCD may lead to disruption of normal cell membrane structure and cell motility dysregulation. In fact, various side effects ranging from moderate to severe have been reported from statin treatment in cancer patients and a significant proportion of individuals show “statin intolerance” depending on the type and dose of statin treatment [30]. Thus, more cancer specific cholesterol targeting strategies are needed for cancer treatment.

**Fig. 6:**
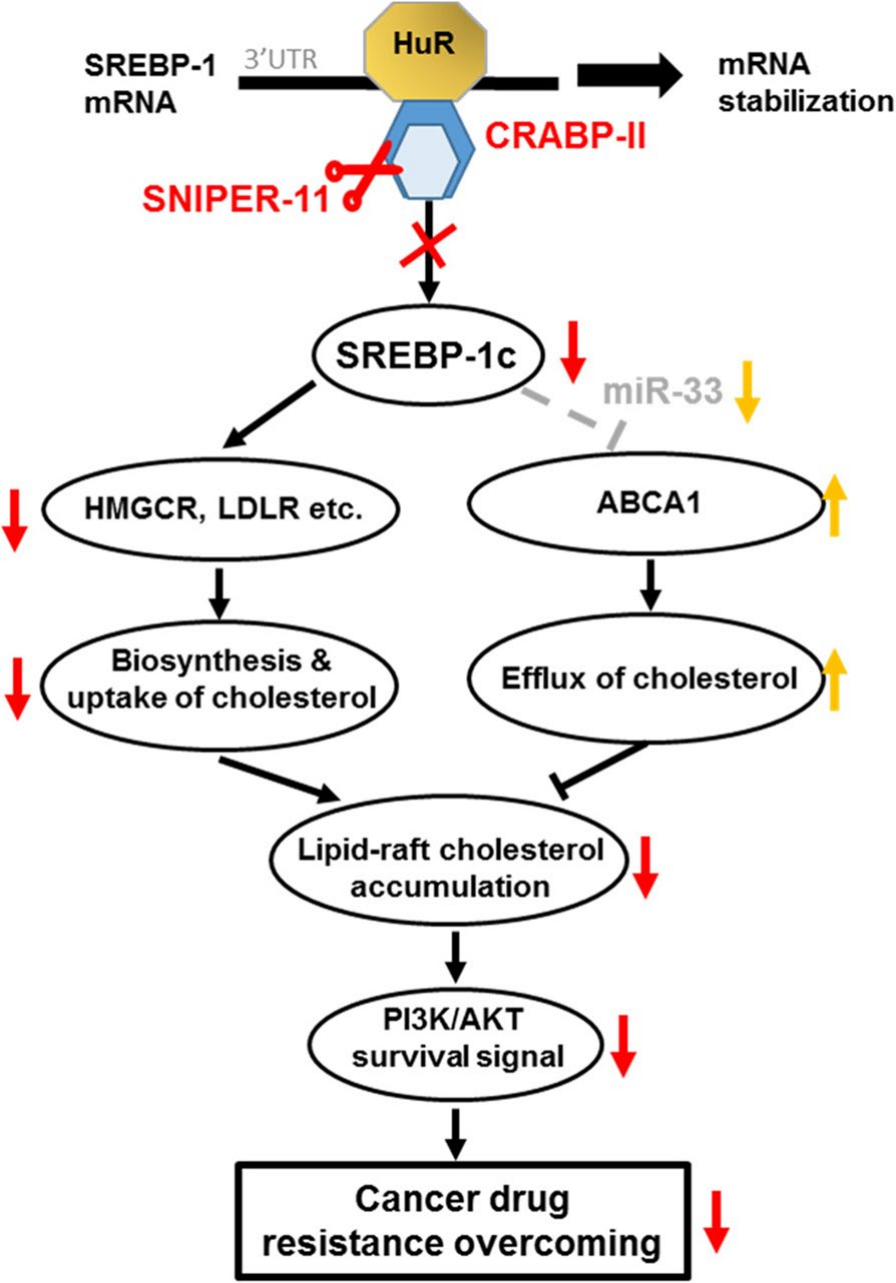
A schematic model showing CRABP-II regulating PDAC cholesterol metabolism and drug resistance. By interacting with HuR, CRABP-II stabilizes the mRNA of SREBP-1c and increases expression of this center lipid metabolic transcription factor. Elevated SREBP-1c activates a cluster of cholesterol metabolic genes such as HMGCR and LDLR, resulting in increased intracellular cholesterol biosynthesis and uptake. SREBP-1c also inhibits the major cholesterol efflux transporter ABCA1 by enhancing miR-33 expression and reduces the intracellular cholesterol removal. Both arms of this machinery lead to the cholesterol accumulation in cell membrane, especially in lipid rafts, thus promoting AKT survival signaling and cancer drug resistance. The protein eraser SNIPER-11 selectively induces CRABP-II degradation in PDAC, hence interrupts CRABP-II/SREBP-1c/raft-cholesterol/AKT axis and overcomes PDAC drug resistance

We previously reported the aberrant expression of CRABP-II in PDAC and identified CRABP-II as a diagnostic biomarker [4]. We also revealed the new role of CRABP-II in the regulation of IL8/MMP2/MMP14 pathway to promote pancreatic cancer migration and invasion [9]. In this study, we further unveiled a novel pathway whereby CRABP-II regulates cholesterol metabolism and PDAC drug resistance. Based on our findings, we propose that CRABP-II forms a complex with HuR, hence enhancing the binding of HuR to the 3’UTR of SREBP-1c mRNA and resulting in the increased stability of SREBP-1c mRNA in cancer cells. This leads to the overexpression of SREBP-1c transcription factor, and its downstream cholesterol metabolic genes, such as *HMGCR* and *LDLR*. SREBP-1c can also inhibit the major cholesterol efflux regulator *ABCA1* via *miR-33* [16]. Together, rewiring of these cholesterol metabolic genes induces the intracellular cholesterol biosynthesis and uptake, decreases the efflux of cholesterol, both in turn leads to the lipid raft cholesterol accumulation (Fig. 6). Further, we found that increased lipid raft cholesterol content in PDAC cells is associated with activated AKT survival signaling and PDAC chemo-resistance, as depletion of CRABP-II not only decreased lipid raft cholesterol level but also inhibited AKT activation and induced cell apoptosis in response to gemcitabine, a process that was reversed by cholesterol or re-expression of CRABP-II. This is a novel function of CRABP-II to promote cancer drug-resistance by interacting with the posttranscriptional regulatory machinery to regulate genes important for cholesterol metabolism and cancer therapy resistance, and is distinct from its well-known role as an RA-carrier to facilitate RAR activation. We hereby have identified CRABP-II as a novel player in pancreatic cancer lipid metabolism, and our observation that CRABP-II is overexpressed in PDAC but not detectable in normal pancreas and chronic pancreatitis justifies CRABP-II as a viable cancer selective target for PDAC therapy.

The canonical ligand of CRABP-II, retinoic acid (RA), has been tested for PDAC treatment in several pilot clinical trials [31–33], but results were either disappointing or showed minimal clinical improvement despite that RA arrests cell cycles, induces differentiation and apoptosis in PDAC cells. This might be attributed to the reduction of retinoids and their receptors including RARs and RXRs in human PDAC tumors [34]. Relative to the low RA concentration in PDAC cells and the microenvironment, highly expressed CRABP-II may be shunted from activating RARs to forming CRABP-II/HuR complex to activate downstream signaling such as promoting lipid raft cholesterol accumulation and promoting tumor chemo-resistance. Our finding that CRABP-II highly expressing tumors are poorly differentiated is consistent with this notion.

In cancer therapy, oncogenic kinase inhibitors such as imatinib and crizotinib have been effective in clinical applications by inhibiting the expression or function of oncogenic proteins. However, such therapy is often hampered by mutation in the respective kinase domains [35]. While genetic knockdown by RNA interference and antisense oligonucleotides are promising cancer treatment options, only limited success has been achieved in clinical applications for siRNA drugs because of their poor cellular delivery and physiological instability [36]. Further, these genetic methods are ineffective in the case of proteins with long half-lives. In this study, we tested an alternative approach to knockdown CRABP-II by inducing its degradation with a synthetic hybrid molecule that specifically crosslinks the ubiquitin ligase cIAP and CRABP-II. We showed that SNIPER-11 is highly effective in reducing CRABP-II level, decreasing cancer cell lipid raft cholesterol accumulation and suppressing ATK signaling, thus enhancing the cytotoxicity of gemcitabine in both PDAC cell lines and orthotopic PDX tumors. In addition, we showed that SNIPER-11 treatment reduced liver metastasis in PDAC bearing mice, consistent with other reports that high cholesterol promotes PDAC metastasis [37] and our previous finding that CRABP-II favored PDAC cell invasion and migration through upregulating IL8/MMP14/MMP2 pathway [9]. Taken together, our results suggest a high potential of SNIPER-11 as an effective PDAC therapeutic option. Compared to conventional kinase inhibitors and siRNAs, this novel “chemical knockdown” approach provides several advantages: It allowed effective ablation of a target protein without modifying the human genome; the treatment is selective and reversible; and it has the same capability of marked protein knockdown efficacy as siRNAs while presenting cellular delivery comparable to small molecular inhibitors. We did not find significant drug toxicity of SNIPER-11 in our study, but the effect of SNIPER-11 on other RA binding proteins such as RARs, and the pharmacologic optimization of dosing and safety would need further investigation.

Although our present study focuses on the role of CRABP-II in raft-cholesterol/AKT pathway, CRABP-II is also involved in the regulation of other lipid metabolism and multiple signaling transductions. For example, our BODIPY staining showed that CRABP-II increased the neutral lipids (cholesterol and triacylglyceride) content in PDAC cells. Indeed, our microarray profiling indicated that the fatty acid synthase gene (*FASN*) was down-regulated in CIIKO. FASN is a downstream target of SREBP-1c and has been associated with PDAC cancer stemness and drug-resistance [38, 39]. Moreover, in addition to AKT pathway, other signaling pathways such as the sonic hedgehog (SHH) and the mucin MUC1 & Src signaling may be impacted by CRABP-II induced lipid raft cholesterol accumulation and contribute to PDAC chemo-resistance [40, 41]. Thus, future studies are warranted to assess the contribution of these signaling molecules and pathways to PDAC drug-resistance regulated by CRABP-II.

## Conclusions

We identified that CRABP-II is a novel upstream regulator of cholesterol metabolism and drug resistance of PDAC and that CRABP-II could serve as a selective target for PDAC therapy. We showed that a small molecule SNIPER-11 effectively degrades CRABP-II and potentiates the sensitivity of PDAC to gemcitabine in both orthotopic PDX and subcutaneous CDX model. Collectively, our work not only reveals an uncanonical function of CRABP-II but also offers a new option for overcoming PDAC drug resistance.

## Supplementary Information

**Additional file 1.**

## Data Availability

All data associated with this study are present in the paper or the Supplementary Materials.

## Abbreviations

CRABP-II: Cellular retinoic acid binding protein II
CIIKO: CRABP-II knockout
CIIOE: CRABP-II overexpression
PDAC: Pancreatic ductal adenocarcinoma
Q-PCR: Quantitative PCR
Co-IP: Co-immunoprecipitation
RNA-IP: RNA immunoprecipitation
PDX: Patient-derived xenografts
SNIPER-11: Specific and nongenetic IAP dependent protein Eraser 11
RA: Retinoic acid
RAR: Retinoic acid receptor
RXR: Retinoid X receptor
3’UTR: The three prime untranslated region
ARE: AU rich element
NSG mice: NOD-SCID-gamma mice
IP administration: Intraperitoneally administration
RBC: Red blood cells
IPA: Ingenuity Pathway Analysis
LC–MS-MS: Liquid chromatography with tandem mass spectrometry
MβCD: Methyl-β-cyclodextrin
EGF: Epidermal growth factor
AKT/PKB: Protein kinase B
PI3K: Phosphoinositide 3-kinases
PDK1: Phosphoinositide-dependent protein kinase-1
mTOR: The mammalian target of rapamycin
GSK3: Glycogen synthase kinase 3
IKK: IκB kinase
SHH: The sonic hedgehog
cIAP: Cellular inhibitor of apoptosis protein 1
PARP: Poly (ADP-ribose) polymerase
SREBP-1: Sterol regulatory element-binding protein 1
SREBF1: Sterol regulatory element binding transcription factor 1
HMGCR: 3-Hydroxy-3-methylglutaryl-CoA reductase
LDLR: Low-density lipoprotein receptor
HMGCS1: 3-Hydroxy-3-methylglutaryl-CoA synthase 1
MVD: Mevalonate diphosphate decarboxylase
INSIG1: Insulin induced gene 1
TMEM97: Transmembrane Protein 97
ABCA1: ATP Binding Cassette Subfamily A Member 1
ADRP: Adipose differentiation-related protein
FASN: Fatty acid synthase
