# Supplementary material for: Targeting CRABP-II overcomes pancreatic cancer drug resistance by reversing lipid raft cholesterol accumulation and AKT survival signaling
Source: J Exp Clin Cancer Res. 2022 Mar 8;41:88. doi: 10.1186/s13046-022-02261-0 (PMC8903155; doi:10.1186/s13046-022-02261-0)
Supplement: Supplementary file 1 — Additional file 1. [file 13046_2022_2261_MOESM1_ESM.pdf]

Table S1. Primer sequences for Q-PCR

Gene Name	Primer Sequence
CRABP2	F: GGTTGGGGAGGAGTTTGAGG<br>R: CAGTTCCCCATCGTTGGTCA
SREBF1	F: GCGCTGCTGACCGACATC<br>R: AGGGAAGTCACTGTCTTGGT
SREBP1a	F: CGGCGCTGCTGACCGACATC<br>R: CCCTGCCCCACTCCCAGCAT
SREBP1c	F: GCGCAGATCGCGGAGCCAT<br>R: CCCTGCCCCACTCCCAGCAT
LDLR	F: GCAGTGGGCGACAGATGTGAA<br>R: GCACGTCTCCTGGGACTCATCA
HMGCR	F: GGGAACCTCGGCCTAATGAA<br>R: CACCACGCTCATGAGTTTCCA
ABCA1	F: TGCAAGGCTACCAGTTACATT<br>R: TTAGTGTTCTCAGGATTGGCT
ACTB	F: CCTCACCCTGAAGTACCCCA<br>R: TCGTCCCAGTTGGTGACGAT

Supplemental Materials

Fig. S1

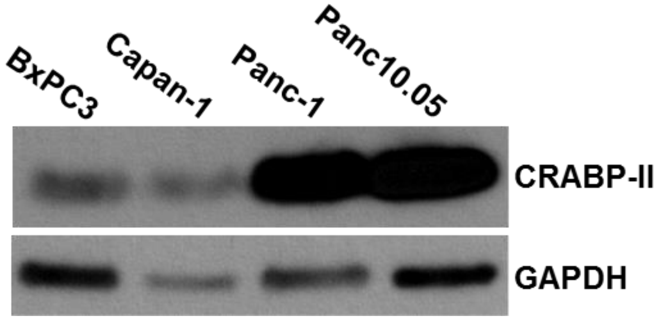

Fig. S1. CRABP-II level in PDAC cell lines assessed by western blot.

Fig. S2

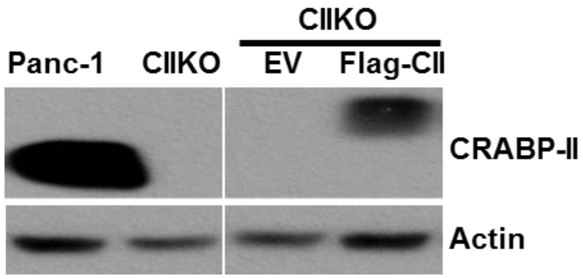

Fig. S2. CRABP-II knockout and re-expression in Panc-1 cells. CRISPR/Cas9 technology was used for knockout and flagged-CRABP-II was re-expressed in CIIKO cells.

Fig. S3

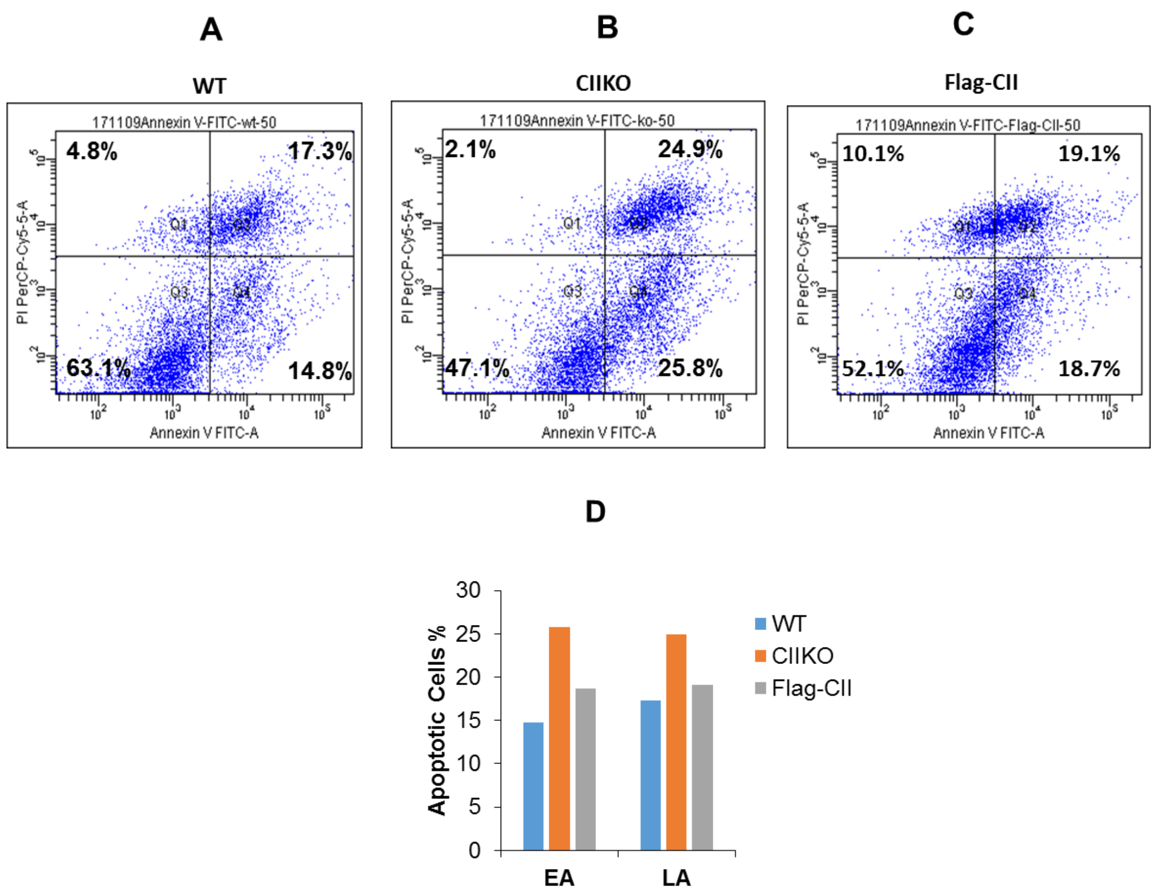

Fig. S3. Apoptosis detection by Annexin V staining. Annexin V staining of WT (**A**), CIIKO (**B**) and Flag-CII expressing (**C**) cells after 24hr treatment with 50 μM of gemcitabine. (**D**) Percentile quantification of early apoptosis (EA) and late apoptosis (LA).

Fig. S4

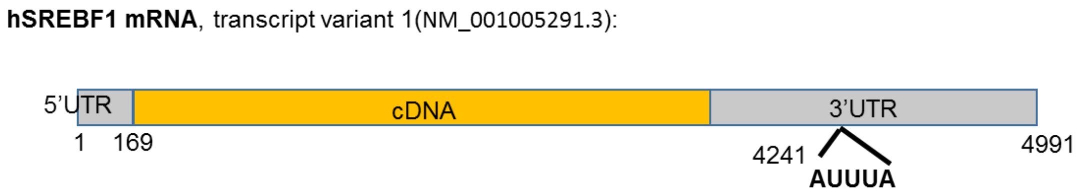

Fig. S4. Putative AREs in the 3'UTR of human SREBP1 mRNA.

Fig. S5

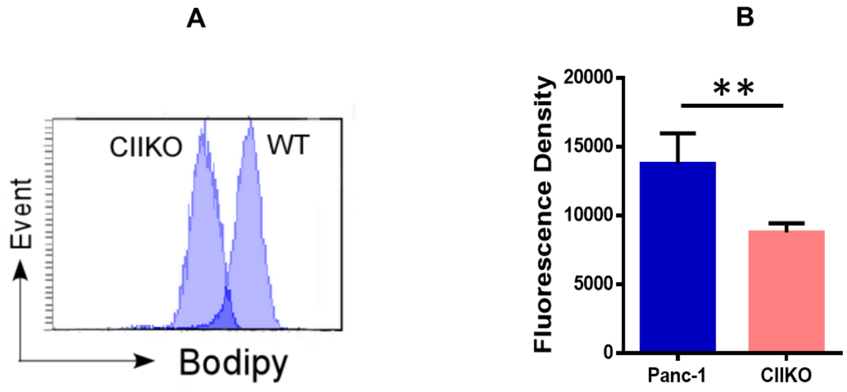

Fig. S5. Intracellular neutral lipids detection by BODIPY staining. Panc-1 wild type control cells and CIIKO cells were stained with BODIPY and analyzed with FACS (**A**). (**B**) Comparison of the means of fluorescence. The experiment was repeated three time and the error bar represents SD. **, $p < 0.01$.

Fig. S6

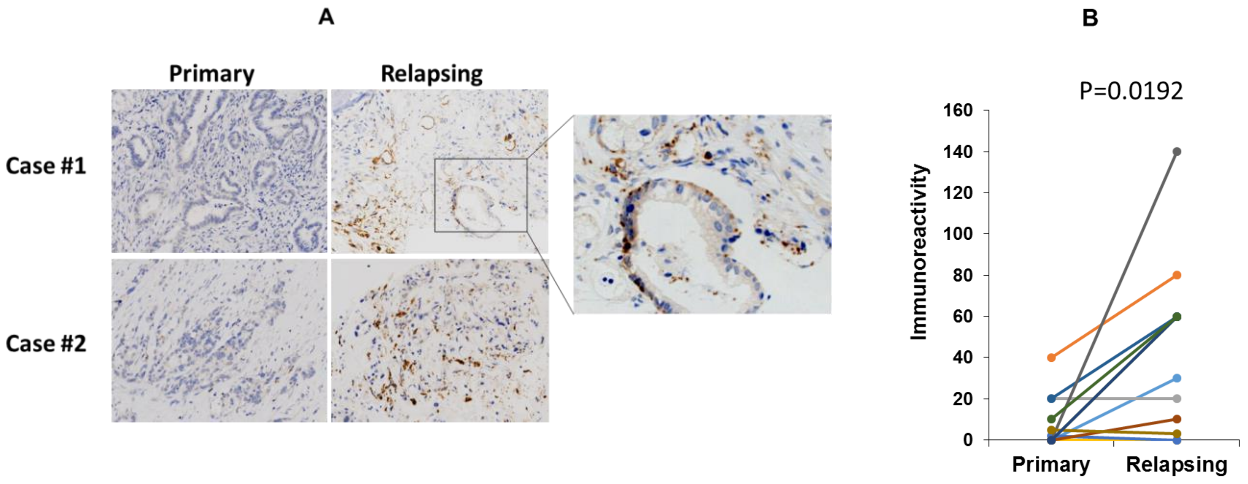

Fig. S6. Comparison of lipid droplet content in primary and relapsing tumors by ADRP staining. (**A**) Positive staining was observed in both cancer epithelia and stromal cells in relapsing tumors while negative or weak staining was in primary tumors. (**B**) Immunoreactivity was calculated by multiplying the percentage of positive epithelium cells and the score of staining intensity (intensity: 0, undetectable; 1, weak; 2, moderate; and 3, strong). Paired sample t-test was used to determine the difference between primary group and relapsing group.

Fig. S7

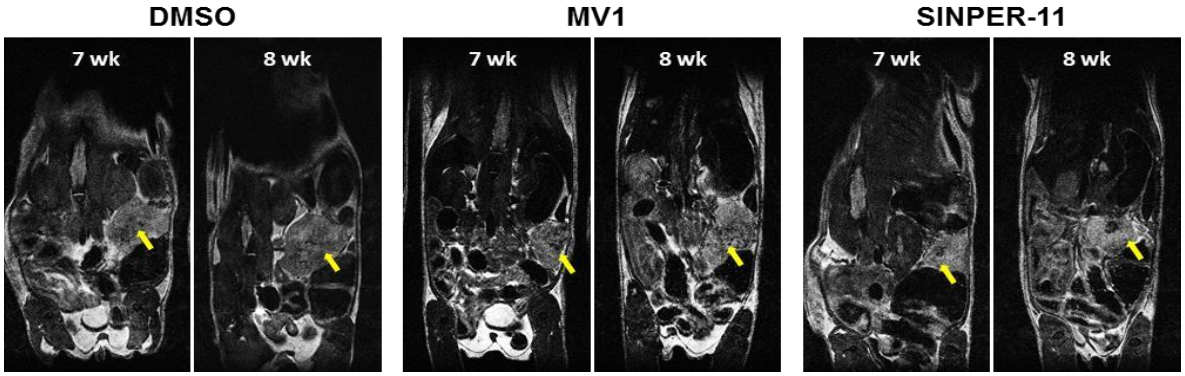

Fig. S7. Tumor repression by SNIPER-11 in pancreas orthotopic PDX assessed by MRI imaging. Limited passaged PDX tumors were digested to single cell suspension and orthotopically injected into NSG mice. At 7 weeks after implantation, the mice were IP administrated with 5 µM of SNIPER-11 or MV1, every two days, DMSO was used as control. Tumor size was monitored using MRI imaging before treatments and after one week treatment.

Fig. S8

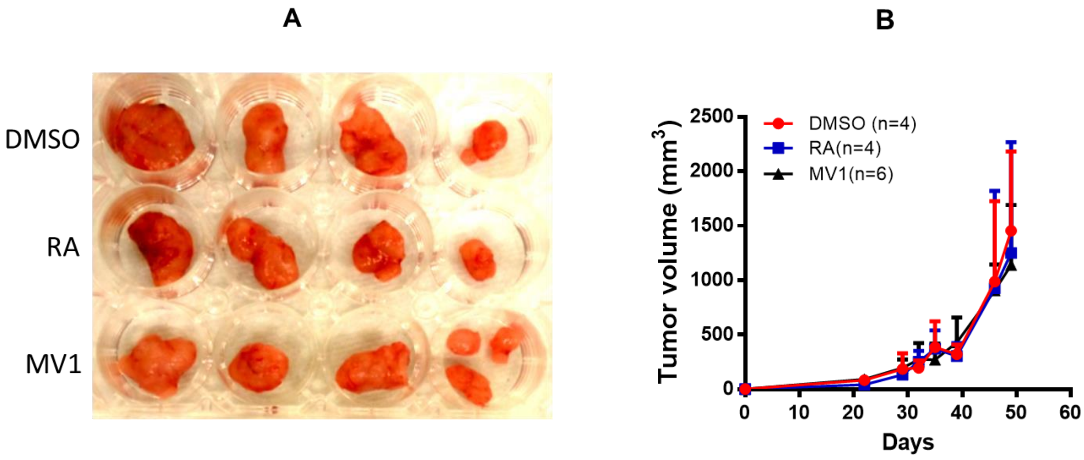

Fig. S8. Tumor growth under RA and MV1 treatment. Limited passaged PDX tumors were dissected to small cubes (1-2 mm^3) and subcutaneously implanted to the back of NSG mice. When the tumor size reached to ~100 mm^3, the tumor bearing mice were grouped and IP administrated with 5 µM of RA and MV1 every two days respectively, DMSO was used as control. All tumors were resected after 3 weeks of treatment (**A**). Tumor growth were measured with calipers and tumor volume = ½(width*width*length) (**B**).

Fig. S9

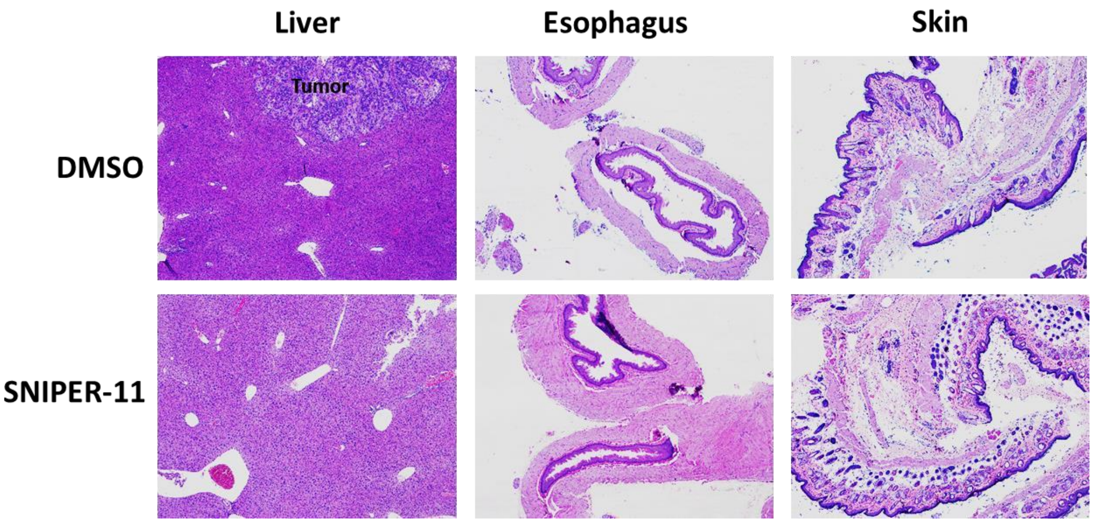

Fig. S9. Histology analysis of liver, esophagus and skin from SNIPER-11 treated mice. Orthotopic PDX bearing NSG mice were treated with 5 μM of SNIPER-11 every two days for 3 weeks and mice were scarified and the CRABP-II expressing normal tissues (esophagus and skin) and mouse livers were fixed for H&E staining.

Fig. S10

DMSO
SN11
Gem
SN11+Gem

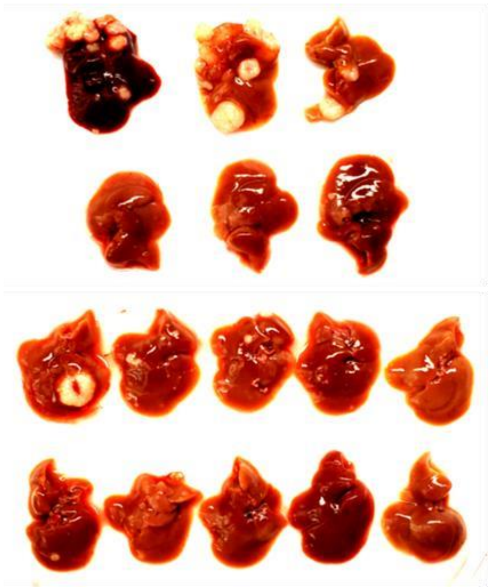

Fig. S10. Liver metastasis of PDAC in orthotopic PDX models. $1x10^6$ PDX single cells were orthotopically implanted to NSG mice. Grouped mice received DMSO, SNIPER-11, gemcitabine or the combination of SNIPER-11 and gemcitabine at 4 weeks after transplantation as described in Methods. After 3 weeks treatment, mice were scarified and the liver metastasis were checked.
